# Horizontal gene transfer in plant microbiomes: integrons as hotspots for cross-species gene exchange

**DOI:** 10.3389/fmicb.2024.1338026

**Published:** 2024-04-29

**Authors:** Timothy M. Ghaly, Michael R. Gillings, Vaheesan Rajabal, Ian T. Paulsen, Sasha G. Tetu

**Affiliations:** ^1^School of Natural Sciences, Macquarie University, Sydney, NSW, Australia; ^2^ARC Centre of Excellence in Synthetic Biology, Sydney, NSW, Australia

**Keywords:** mobile genetic elements, plant-associated bacteria, phyllosphere, rhizosphere, plant-bacterial interactions, niche adaptation, plant growth-promoting, phytopathogen

## Abstract

Plant microbiomes play important roles in plant health and fitness. Bacterial horizontal gene transfer (HGT) can influence plant health outcomes, driving the spread of both plant growth-promoting and phytopathogenic traits. However, community dynamics, including the range of genetic elements and bacteria involved in this process are still poorly understood. Integrons are genetic elements recently shown to be abundant in plant microbiomes, and are associated with HGT across broad phylogenetic boundaries. They facilitate the spread of gene cassettes, small mobile elements that collectively confer a diverse suite of adaptive functions. Here, we analysed 5,565 plant-associated bacterial genomes to investigate the prevalence and functional diversity of integrons in this niche. We found that integrons are particularly abundant in the genomes of Pseudomonadales, Burkholderiales, and Xanthomonadales. In total, we detected nearly 9,000 gene cassettes, and found that many could be involved in plant growth promotion or phytopathogenicity, suggesting that integrons might play a role in bacterial mutualistic or pathogenic lifestyles. The rhizosphere was enriched in cassettes involved in the transport and metabolism of diverse substrates, suggesting that they may aid in adaptation to this environment, which is rich in root exudates. We also found that integrons facilitate cross-species HGT, which is particularly enhanced in the phyllosphere. This finding may provide an ideal opportunity to promote plant growth by fostering the spread of genes cassettes relevant to leaf health. Together, our findings suggest that integrons are important elements in plant microbiomes that drive HGT, and have the potential to facilitate plant host adaptation.

## Introduction

Plants are readily colonised by diverse bacteria, many of which play important roles in plant health. These bacteria can adopt a range of lifestyles, from pathogenic to commensal to mutualistic. Collectively, however, plant microbiomes often confer a range of plant growth-promoting traits (PGPTs) that increase plant fitness—enhancing nutrient uptake, suppressing diseases, and mitigating the impacts of abiotic stress (Trivedi et al., 2020). Understanding the ecology and evolution of plant microbiomes is essential for fundamental knowledge of plant biology, and also as a means to promote sustainable agriculture and ecosystem health (Bardgett and Van Der Putten, 2014; Bender et al., 2016; Wubs et al., 2016).

One important aspect of plant microbial ecology is horizontal gene transfer (HGT), which is defined as the transfer of DNA between different organisms. HGT can have negative fitness effects on recipient bacteria (Ghaly and Gillings, 2022), but can also be a powerful evolutionary driver (Soucy et al., 2015). HGT can bestow new traits upon recipients, acting as an important source of genotypic and phenotypic innovation (Pál et al., 2005). Interestingly, bacterial HGT can have substantial implications for plant health. Indeed, many bacterial genes that facilitate symbioses with plants are mobile, with acquisition of these genes often determining the mutualistic or pathogenic lifestyles of the recipient bacteria (Lima et al., 2008; Vanga et al., 2015; De Assis et al., 2022; Wardell et al., 2022). Thus, identifying both the bacterial species and genetic elements involved in HGT is an essential step towards a more comprehensive understanding of plant microbiome dynamics and plant health.

Integrons are genetic elements known to facilitate horizontal gene transfer among bacteria by functioning as site-specific gene capture and expression systems (Mazel, 2006; Gillings, 2014; Ghaly et al., 2021a). They capture, excise and shuffle small mobile genetic elements, known as gene cassettes, within integron platforms (Stokes and Hall, 1989). Integron gene cassettes generally consist of an open reading frame (ORF) and a cassette recombination site (*attC*). Integration is mediated by the integron integrase (IntI), a site-specific recombinase, encoded by integrons (Collis et al., 1993). The acquisition of novel gene cassettes, which are extremely diverse and abundant in the environment (Ghaly et al., 2022a), can lead to rapid adaptation in bacteria (Escudero et al., 2015). Although many of their functions are unknown, cassettes often encode proteins involved in stress tolerance, cell interactions, and niche adaptation (Ghaly et al., 2023). The wide taxonomic distribution of integrons, which occur among diverse bacterial and archaeal phyla (Cury et al., 2016; Néron et al., 2022; Ghaly et al., 2022b), means that they can facilitate horizontal transfer of such traits across broad phylogenetic boundaries (Ghaly et al., 2022b).

Integrons are known to occur among diverse plant-associated bacteria (Gillings et al., 2005; Ghaly et al., 2021b), and the microbiomes of different plant species harbour diverse and distinct suites of gene cassettes (Rajabal et al., 2023). Interestingly, the rhizoplane of different plant species exhibit greater distinction in their gene cassette profiles than their microbial composition (Rajabal et al., 2023), suggesting that gene cassettes play a role in bacterial interactions with specific plant species.

Here, we examined the distribution and functional cargo of integrons in the genomes of 5,565 plant-associated bacterial isolates (Patz et al., 2021). We found that integrons are common among plant microbiomes, and often encode putative bacterial-plant interaction traits. We note that there is a significant difference in the functional profiles of gene cassettes between rhizosphere- and phyllosphere-associated strains. We also find that integrons are hotspots of cross-species HGT. The frequency of HGT appears to be enhanced in the phyllosphere relative to the rhizosphere. Together, our findings suggest that integrons are important elements in plant microbiomes, and that they can facilitate plant host adaptation and HGT.

## Methods

### Integron screening

Data for plant-associated bacteria were obtained from PLaBA-db (Patz et al., 2021), which comprises 5,565 plant-associated bacterial isolate genomes, along with metadata, including plant isolation site. Assembled genomic sequences from all bacterial isolates listed in PLaBA-db were downloaded from IMG/M (Chen et al., 2023). Genomes were screened for integrons using IntegronFinder v2.0rc6 (Cury et al., 2016; Néron et al., 2022) [parameters: --local-max --cpu 24 --gbk]. Complete integron sequences, along with *attC* and integron integrase (IntI) sequences, were extracted from IntegronFinder’s GenBank-formatted output using the *gbfcut* tool from the FAST software package (Lawrence et al., 2015), with parameters [−k integron], [−k *attC*], and [−k integrase], respectively. Open reading frames (ORFs) within extracted integron sequences were predicted using Prodigal v2.6.3 in metagenome mode [parameters: -q -p meta]. Putative IntIs were identified via protein sequence homology to IntegronFinder’s IntI annotations using BLASTp, with cut-offs of 95% amino acid identity and 95% query cover. Identified IntI sequences were separated from the remaining cassette-associated proteins for downstream analyses.

#### Functional annotations of cassette-associated proteins

Functional annotations of cassette-associated proteins were made using eggNOG-mapper v2.1.9 (Cantalapiedra et al., 2021), based on eggNOG v5.0.2 orthology data (Huerta-Cepas et al., 2018). eggNOG-mapper sequence searches were performed using DIAMOND v2.0.15 (Buchfink et al., 2021). Given their high prevalence among gene cassette functional annotations (Ghaly et al., 2023), categories of Clusters of Orthologous Genes (COGs) (Galperin et al., 2020) assigned to toxin-antitoxin (TA) components were re-categorised as “Toxin-antitoxin systems.” TA system genes were identified based on the following eggNOG-mapper annotation terms: “toxin,” “antitoxin,” “addiction module,” “plasmid maintenance system killer,” “plasmid stabilization,” “antidote,” “TA system,” “antitox,” and “post-segregation.” In addition, transposases, which often target gene cassette arrays for insertion (Tetu and Holmes, 2008), and are thus included in the identified cassette-associated proteins, were re-categorised as “Transposases.” Transposases were also assigned to their insertion sequence (IS) families based on top BLASTp hits to the ISfinder database (Siguier et al., 2006). Additionally, we screened for putative antibiotic resistance gene cassettes using NCBI’s AMRFinderPlus (Feldgarden et al., 2021), with default parameters.

We identified cassette-associated proteins that could confer plant-growth promoting traits (PGPTs) using a list of KEGG Ortholog (KO) identifiers (Kanehisa and Goto, 2000) from the plant growth promotion traits ontology (Patz et al., 2021; Ashrafi et al., 2022). From this list of traits, only KOs from the following subclasses were used: “Bio-fertilization,” “Stress_control|biocontrol,” “Bio-remediation,” “Phytohormone|plant_signal_production,” “Plant_immune_response_stimulation,” and “Colonising_plant_system.” We screened all cassette-associated proteins for these KOs, using KofamScan v1.3.0 (Aramaki et al., 2019). Only KO assignments that had a score higher than the model-specific threshold (Aramaki et al., 2019) were retained.

Gene cassettes encoding putative plant pathogenic effector proteins were identified using a combination of EffectiveT3 (Eichinger et al., 2015) and Bastion3 (Wang et al., 2018) predictions, each of which identify possible secreted effectors from protein sequence features. We considered only agreements in prediction from the two tools as effector proteins.

#### Identification of horizontal gene cassette transfer

To identify putative horizontal gene transfer (HGT) events, we screened for instances of different species harbouring gene cassettes that shared 100% nucleotide identity. Cases of identical cassettes shared between strains of the same species were excluded, to ensure vertical transmission was not misidentified as an HGT event. Cassette homology was identified using BLASTn all-*vs*-all searching. Self-hits and inverted query-subject pairings were removed. Of the remaining hits, only those with 100% nucleotide identity and 100% query cover were retained to ensure maximum stringency in calling HGT events.

#### IntI and *attC* trees

To determine *attC* similarity, we used a structural, rather than a sequence homology approach. This is because the site-specificity of *attC* recombination sites relies on their folded secondary structures rather than their DNA sequence alone (Nivina et al., 2020). The relationship between *attC*s from different taxa was determined using hierarchical-clustering based on similarity of their folded structures, thus generating a structure-based *attC* tree (Ghaly et al., 2021b). However, the computational time for such an approach, which relies on pairwise structural alignments of each *attC* with every other *attC*, increases exponentially with increasing number of *attC* sequences. Therefore, to reduce this computational cost, we selected the ten most representative *attC*s from each bacterial clade (either at genus or family level, depending on *attC* abundance). To identify the most representative *attC*s, we first generated within-clade structural alignments of non-redundant sets of *attC*s using LocARNA v1.9.2.1 (Will et al., 2007, 2012) [parameters: mlocarna --stockholm --threads 8]. These structural alignments were used to build covariance models using the *cmbuild* and *cmcalibrate* tools from the Infernal v1.1.2 software package (Nawrocki and Eddy, 2013), with default parameters. Each covariance model was used to search the clade-specific sets of *attC*s using Infernal’s *cmsearch* tool [parameters: --notrunc] to identify which *attC*s best fit their clade-specific model. The ten top scoring *attC*s were then clustered using RNAclust v1.3, which builds on LocARNA to construct a WPGMA (weighted pair group method with averaging) tree from the *attC* structural alignment.

To infer the IntI phylogeny, we first aligned protein sequences using MUSCLE v5.1 [parameters: -align -threads 28]. The resulting multiple sequence alignment (MSA) was trimmed using trimAl v1.4.rev15 (Capella-Gutiérrez et al., 2009), applying the *gappyout* model. A maximum-likelihood phylogenetic tree was generated from the trimmed MSA using IQ-TREE 2.2.0-beta (Minh et al., 2020) with 1,000 bootstrap replicates and applying the Q.pfam+F + R7 amino acid substitution model, determined as the best fitting model by ModelFinder (Kalyaanamoorthy et al., 2017) [parameters: iqtree2-B 1000-alrt 1,000-T 12]. Both the *attC* and IntI trees were visualised and annotated using the ggtree v2.4.2 (Yu et al., 2017) and ggtreeExtra v1.7.0.990 (Xu et al., 2021) R packages.

We performed co-phylogenetic analyses to assess the level of incongruence between IntI, *attC* and host trees. First, to generate the host tree, we constructed a whole genome phylogeny using a concatenated set of 120 marker proteins (Parks et al., 2017). These markers were identified and aligned using the *identify* and *align* commands within GTDB-Tk v2.1.1 (Chaumeil et al., 2022), respectively, with default parameters. A phylogenetic tree was generated from the concatenated marker MSA using FastTree v2.1.11 (Price et al., 2010), applying the LG amino acid substitution model (Le and Gascuel, 2008). LG was determined to be the best fit out of FastTree2’s available models (WAG, LG, JTT) using ModelFinder [parameter: -m TESTONLY -mset WAG, LG, JTT]. To visually assess incongruence between IntI, *attC* and host trees, we constructed pairwise tanglegrams between each of the trees using the R package Rtapas v1.1.1 (Llaberia-Robledillo et al., 2023). To quantitatively assess the level of incongruence between trees, we calculated the distance between tree topologies using the nPH85 metric (Geoghegan et al., 2017). We calculated nPH85 using the *dist.topo.normalised* function from the R package NELSI v0.21 (Ho et al., 2015), with 10,000 topology randomisations.

## Results and discussion

### Integrons in plant-associated bacteria

**Figure 1:**
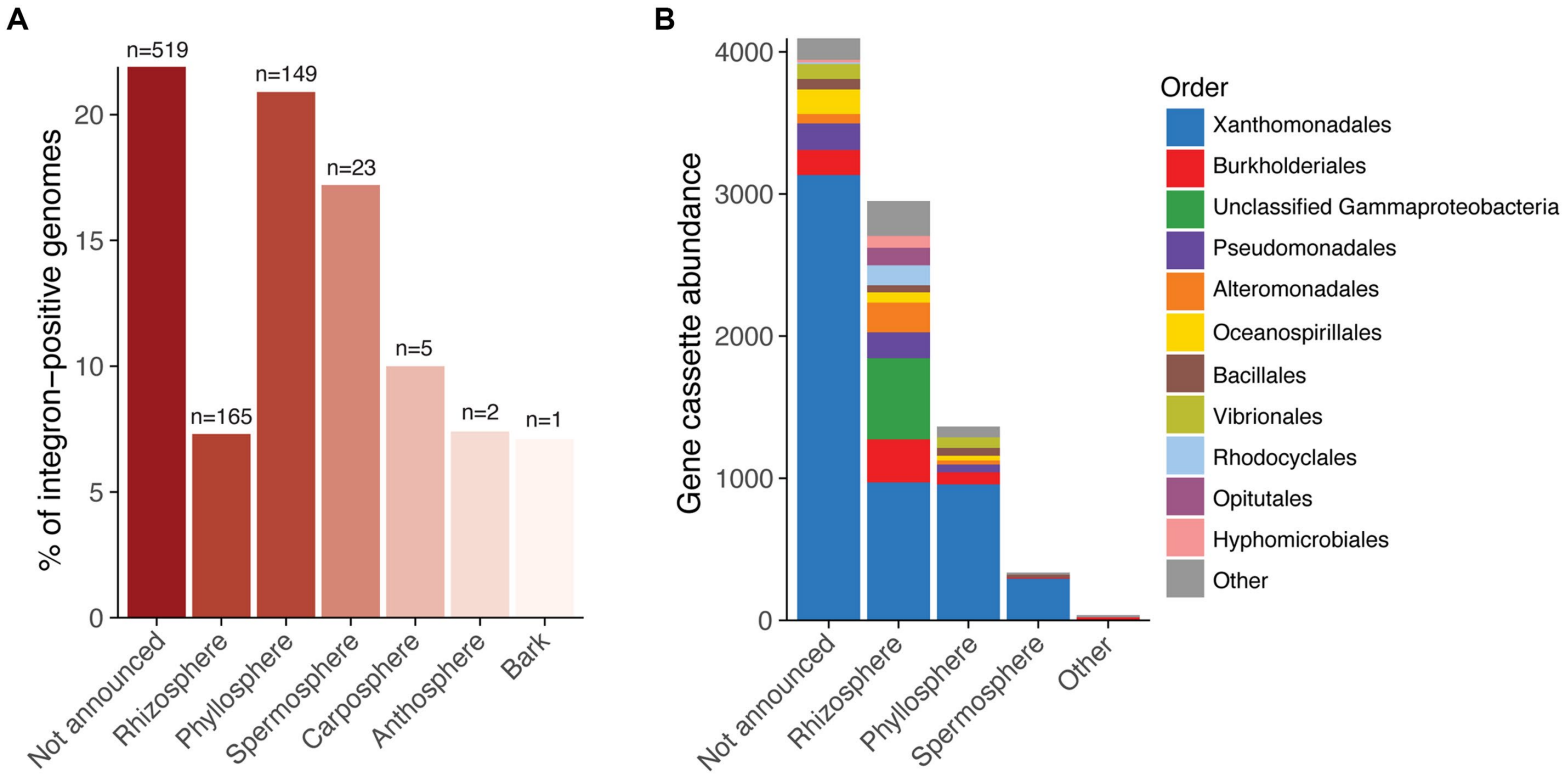
Frequency of integrons in plant-associated bacterial genomes. **(A)** Percentage of bacterial genomes that carry integrons categorised by plant isolation site. The red colour scale indicates the number of genomes with integrons for each plant site, ranging from one genome (light red) to more than 500 genomes (dark red). The total number of genomes containing integrons is displayed above each bar. **(B)** Stacked bar chart showing gene cassette abundance for each plant site. Colours indicate the order-level taxonomy of the bacterial genomes in which the gene cassettes reside.

Integrons are genetic elements that have the capacity to confer diverse adaptive functions and drive horizontal gene transfer (HGT). To investigate their prevalence and potential roles in promoting HGT within plant microbiomes, we screened 5,565 isolate genomes of plant-associated bacteria for integrons. The genomes, sourced from PLaBA-db (Patz et al., 2021), were largely comprised of bacteria from the orders Hyphomicrobiales (predominantly Rhizobiaceae, Bradyrhizobiaceae, and Phyllobacteriaceae), Pseudomonadales, Xanthomonadales, Bacillales, Enterobacterales, Micrococcales, and Burkholderiales (Supplementary Figure S1). Overall, we found that 15.5% of isolate genomes (*n* = 864) were positive for integrons, although this ranged from 7–22%, depending on plant isolation site (Figure 1A; Supplementary Table S1). In total, these integrons comprised 305 integron integrases (IntIs) and 7,445 gene cassettes, collectively encoding 8,778 cassette-associated proteins. The majority of integron gene cassettes were derived from the order Xanthomonadales (Figure 1B), which made up 10.9% of the complete genome database, but 58.7% of the set of integron-positive genomes. Integrons have been previously reported to be an important source of genomic diversity among Xanthomonadales (Gillings et al., 2005), and many integron gene cassettes of clinical relevance are predicted to have originated from this bacterial order (Ghaly et al., 2021b). Their overrepresentation among integron-positive genomes in this dataset suggest that Xanthomonadales are an important source of gene cassettes within plant microbiomes.

### Gene cassette functions differ between the phyllosphere and rhizosphere

**Figure 2:**
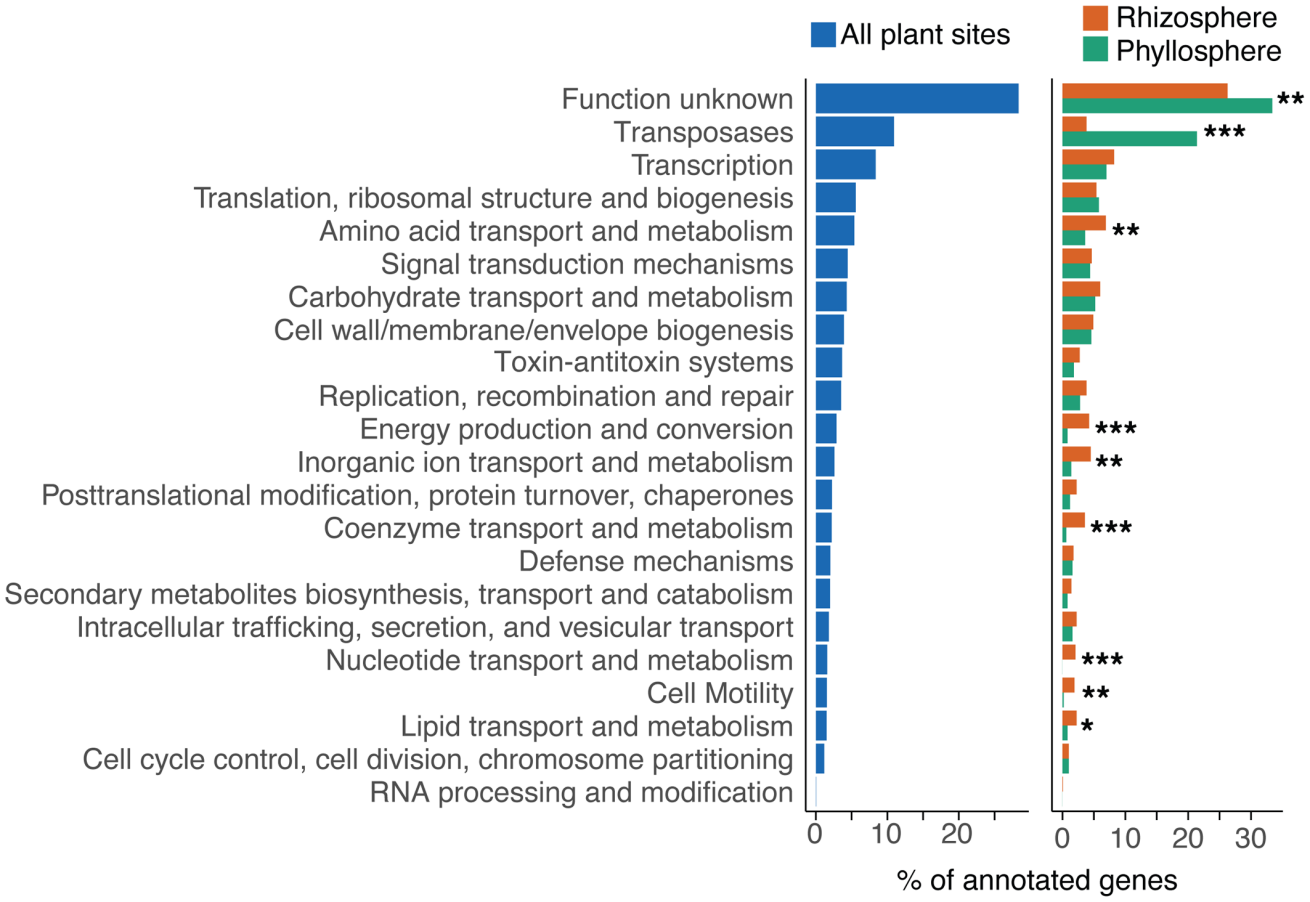
Functional categories of cassette-associated proteins. Functional categories were based on eggNOG-mapper assignments of COG categories for the complete dataset (left), and for rhizosphere and phyllosphere cassette proteins separately (right). Genes annotated as transposases, or as part of toxin-antitoxin systems, were re-assigned their own categories. Asterisks indicate significant difference in the proportion of proteins assigned to a category between the rhizosphere and phyllosphere, as determined by pairwise Fisher’s exact tests, where * signifies *p* < 0.05, ** signifies *p* < 0.01, and *** signifies *p* < 0.001.

Functional categorisations, based on eggNOG-mapper assignments of Cluster of Orthologous Genes (COG) categories (Galperin et al., 2020), could be made for 33.3% (*n* = 2,927) of cassette-associated proteins. Of these, however, 28.4% were assigned to the COG category “Function unknown” (Figure 2). Consequently, about 25% of cassette-associated proteins could be assigned an identifiable putative function.

We found that the functional profile of cassettes recovered from rhizosphere resident bacteria (33.6% of recovered cassettes) differed from those of phyllosphere bacteria (15.4% cassettes) (Figure 2). The rhizosphere was associated with a significant increase (Pairwise Fisher’s Exact Tests, *p* < 0.05) in the proportion of proteins assigned to the COG categories “Energy production and conversion”, “Coenzyme transport and metabolism”, “Nucleotide transport and metabolism”, “Amino acid transport and metabolism”, “Inorganic ion transport and metabolism”, “Lipid transport and metabolism”, and “Cell motility”. Together, this trend suggests that there is selection in the rhizosphere for gene cassettes involved in transport and metabolism of diverse substrates. We suspect that this might enhance bacterial adaptation to plant roots, perhaps dealing with the complex biochemistry of root exudates, and a greater diversity of microbial interactions (Bulgarelli et al., 2013).

Conversely, the phyllosphere was associated with a significant increase (Pairwise Fisher’s Exact Tests, *p* < 0.01) in the categories “Transposases” and “Function unknown”. These transposases belonged to a range of insertion sequence (IS) families, however, most belonged either to the IS5 (49%), IS3 (23%) or IS110 (IS1111 group; 10%) families. The invasion of transposable elements into integrons, particularly those targeting *attC* sites for insertion, such as the IS1111 group, has previously been reported (Tetu and Holmes, 2008; Post and Hall, 2009). However, to the best of our knowledge, there has been no report of the frequency of such events being influenced by environment type. It is possible that transposase activity is higher in the phyllosphere due to exposure to more acute fluctuations in temperature and UV irradiation (Hirano and Upper, 2000). Both of these parameters are known to upregulate transposase activity (Vandecraen et al., 2017), and thus might explain a greater frequency of transposase invasion of integrons in the phyllosphere.

### Integrons as a source of plant-bacterial interaction traits

**Figure 3:**
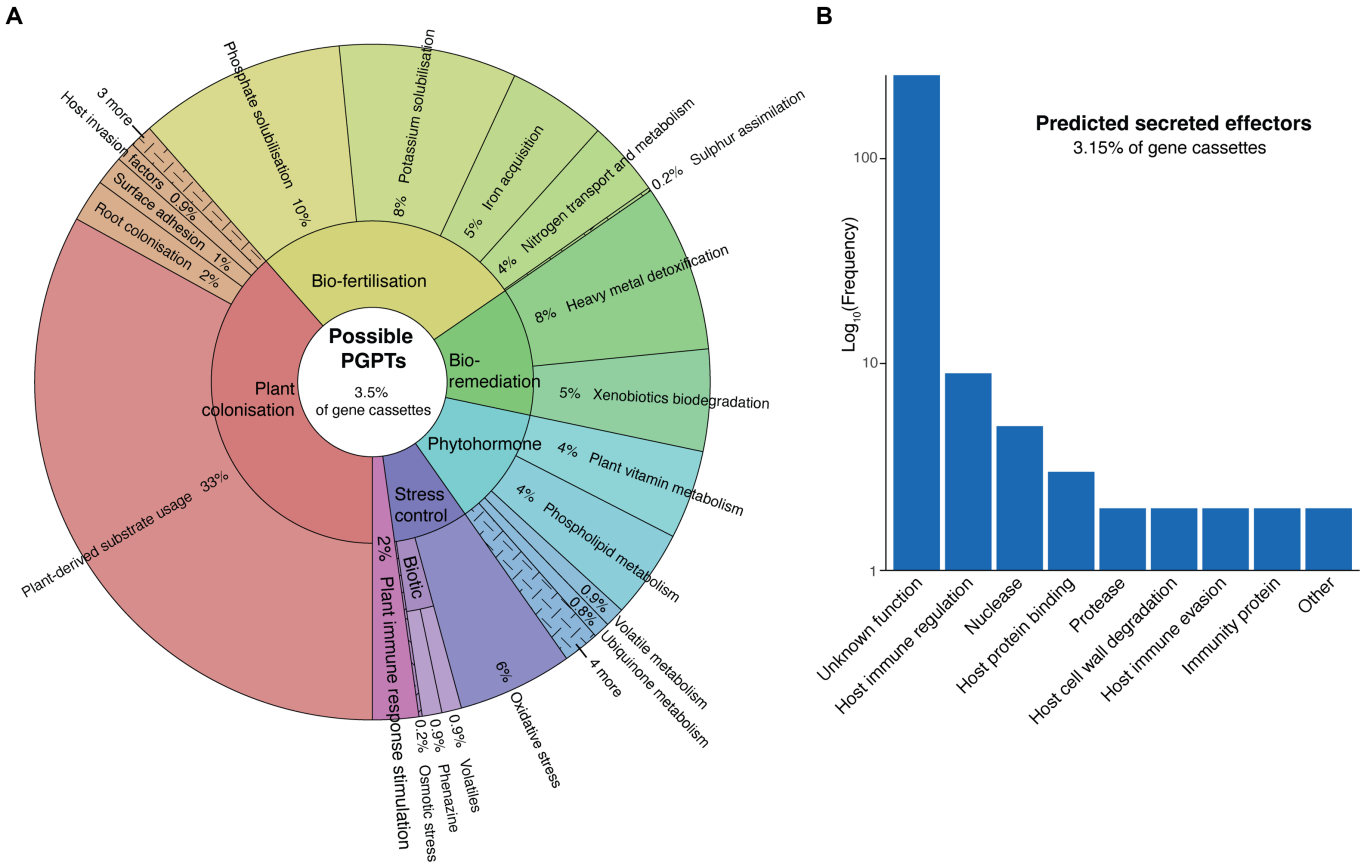
Plant interaction traits encoded by cassette-associated proteins. **(A)** Krona plot (Ondov et al., 2011) showing the functional profile of gene cassettes that confer possible plant growth-promoting traits (PGPTs). **(B)** Abundance of functional annotations assigned to gene cassettes encoding predicted plant pathogenic effector proteins.

We identified a diverse suite of gene cassettes potentially encoding traits involved in plant interaction. Using a curated set of KEGG orthology identifiers (KOs), we could assign 3.5% (*n* = 309) of cassette proteins as conferring possible plant growth-promoting traits (PGPTs) (Figure 3A). These genes are predicted to be involved in plant colonisation, bio-fertilisation, bio-remediation of heavy metals and xenobiotic compounds, phytohormone metabolism, stress control, and stimulation of plant immune responses. It should be noted that many of these traits are not necessarily exclusive to plant growth promotion, or even plant-bacterial interactions, as they can be relevant to bacterial fitness across a range of different environments. Some of these may also be involved in plant pathogenicity. Nevertheless, all of these traits have been implicated in plant growth promotion, and are enriched in the genomes of plant-associated bacteria relative to other environments (Patz et al., 2021).

The most abundant PGPT category was plant-derived substrate usage (plant colonisation), comprising 33% of all PGPT cassettes (Figure 3A). These were largely involved in the transport and metabolism of plant-derived carbohydrates, organic acids, phenolic and terpenoid compounds, amino acids, nucleosides and lipids. The majority (63%) of these cassettes were derived from the rhizosphere. This percentage of rhizosphere-associated cassettes rose to 90% when considering those only from genomes with an announced plant isolation site. This mirrors the findings from the COG analysis (Figure 2), which showed a significant enrichment of such traits in the rhizosphere. These findings add to the growing evidence that integrons can facilitate bacterial-host interactions, along with host adaptation and colonisation (Ghaly et al., 2023).

Conversely, we found that 3.15% (*n* = 277) of cassette proteins were potential determinants of pathogenicity. We identified these as putative secreted effector proteins using an ensemble of sequence feature predictors (Eichinger et al., 2015; Wang et al., 2018). Effectors are secreted virulence proteins injected into host cell cytoplasm by Gram-negative secretion systems to facilitate infection of their hosts (Alfano and Collmer, 2004). Most of these predicted cassette-encoded effectors could not be assigned a function, however, the remaining were associated with a broad range of plant pathogenic traits, including host immune regulation, host immune evasion and protein binding, immunity proteins, and the degradation of host nucleic acids, proteins, and cell walls (Figure 3B). The largest of the known function categories was host immune regulation, which predominantly included Toll/interleukin-1 receptor (TIR) domain proteins and transcription activator-like (TAL) effectors. TIR domains are common among both plants and phytopathogenic bacteria (Sarris et al., 2016), and effectors encoding TIR domains are known to interfere with host immune signalling, suppressing their innate immunity (Cirl et al., 2008). TAL effectors, which are mostly associated with *Xanthomonas* pathovars, are transcription factors that regulate host gene expression. TAL effectors play an important role in pathovar-host specificity (Mak et al., 2013), which provides further evidence that integrons might be involved in the adaptation of bacterial pathogens to specific plant host species (Gillings et al., 2005).

Given the established role of integrons in conferring antibiotic resistance in clinical settings (Souque et al., 2021; Corno et al., 2023; Hipólito et al., 2023), we also screened for antibiotic resistance gene (ARG) cassettes among plant-associated integrons. Of the nearly 9,000 gene cassettes, however, only 16 (0.18%) of these were identified as putative ARGs. These largely encoded predicted determinants of resistance to aminoglycosides, beta-lactams, and trimethoprim. In contrast, ~33% of gene cassettes associated with clinical integrons are putative ARGs (Ghaly et al., 2022a). This highlights the role of integrons in maintaining environment-specific suites of genes that confer adaptive traits.

### Frequency of horizontal gene cassette transfer differs by plant site

**Figure 4:**
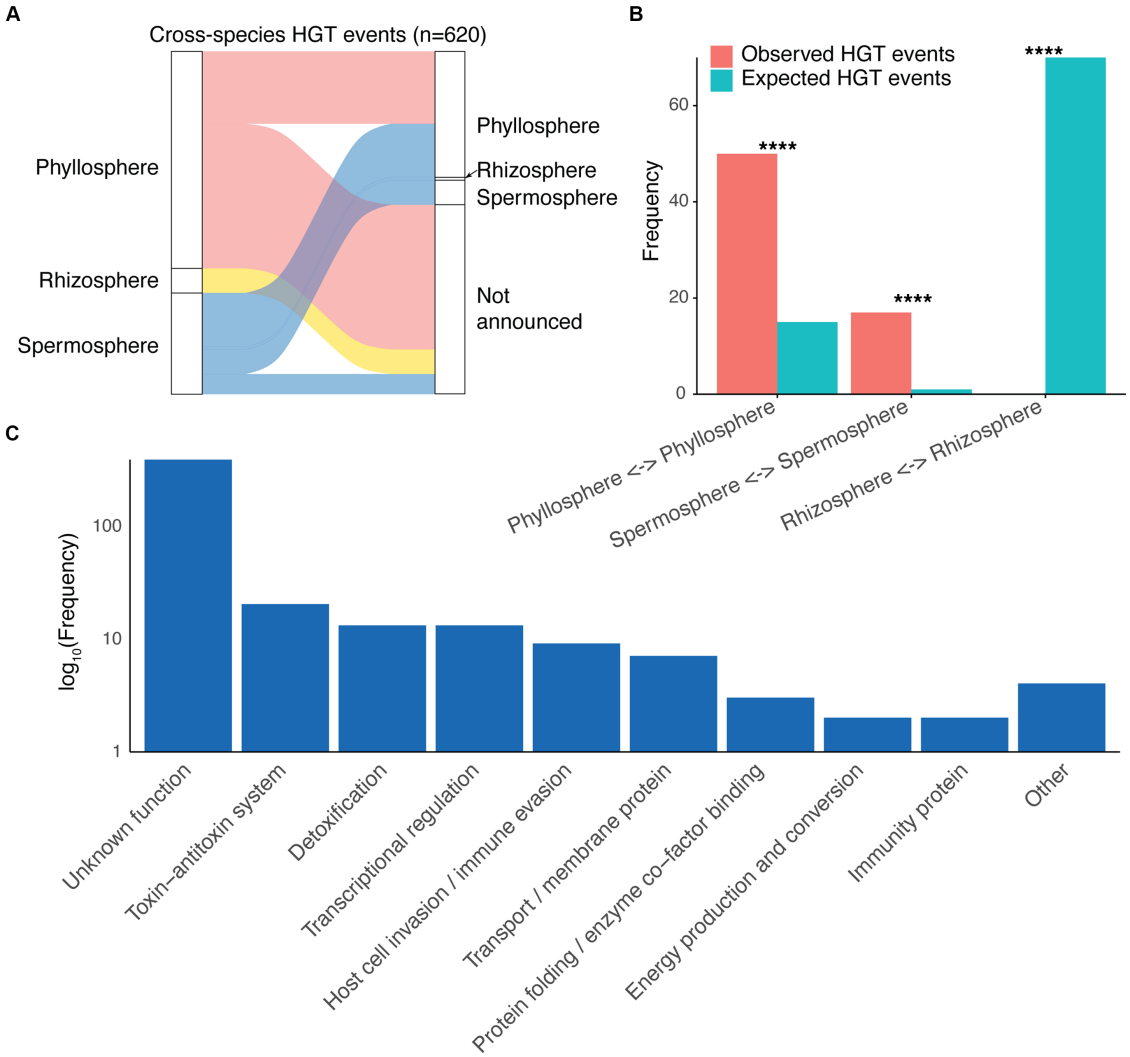
Cross-species horizontal gene cassette transfer. **(A)** A Sankey diagram showing cross-species horizontal gene transfer (HGT) among bacteria isolated from different plant sites. For clarity, cases of HGT in which both species had plant isolation sites as “Not announced” are omitted. **(B)** Observed abundance of cross-species HGT events within each plant site compared to HGT counts that would be expected probabilistically based on cassette abundances for each plant site. All comparisons were statistically significant (*p* < 0.001), as determined by Fisher’s exact tests. **(C)** Abundances (log_10_ scale) of different functions assigned to horizontally transferred gene cassette proteins.

Given their mobile nature and their potential for facilitating microbial adaptation, we examined potential horizontal gene transfer (HGT) of cassettes between different species of plant-associated bacteria. Gene cassettes sharing 100% nucleotide identity and resident in different bacterial species were considered to be horizontally transferred. Cases of identical cassettes shared between strains of the same species were not considered, ensuring vertical transmission was not misidentified as horizontal transmission. We detected 620 HGT events, involving 464 gene cassettes (5.5% of all gene cassettes). As expected, we found that HGT events occurred between species associated with the same plant site, with the notable exception of the spermosphere (Figure 4A). This is not surprising given that bacteria associated with plant seeds (i.e., the spermosphere), may eventually colonise either the plant leaves or roots, post germination.

Interestingly, however, we note a difference in HGT frequency between plant sites (Figure 4B). There was a significantly greater frequency of HGT events within the phyllosphere and spermosphere than would be expected probabilistically based on cassette abundances for each plant site (Fisher’s Exact Tests, *p* < 0.001; Figure 4B). Expected HGT events were calculated based on the null hypothesis that there is no difference in HGT rates between plant sites, and thus the number of HGT events would be expected to be proportional to the number of cassettes detected from each plant site. Conversely, there were no HGT cases between different species within the rhizosphere, despite the expected number of HGT events to be the greatest of all plant sites (*n* ≈ 70) (Figure 4B). This suggests that gene cassettes are more mobile within the phyllosphere, and less so in the rhizosphere. To the best of our knowledge, this has not been previously reported. These findings, together with the frequency of transposase invasions, suggests that there may indeed be greater movement of mobile genetic elements more broadly in the phyllosphere compared to the rhizosphere, although experimental evidence, including studying HGT *in situ* (Sørensen et al., 2005), would be needed to confirm this.

Horizontally transmitted cassettes were mostly of unknown functions, although those with characterised functions spanned a range of categories, with many putatively involved in plant-interactions (Figure 4C). Recent studies have shown that many plant symbiosis genes are mobile, located on plasmids as well as integrative and conjugative elements (ICEs) (Wardell et al., 2022). Interestingly, acquisition of these mobile genes can determine the symbiotic lifestyle (pathogenic or beneficial) of plant-associated bacteria (Lima et al., 2008; Vanga et al., 2015; De Assis et al., 2022; Wardell et al., 2022). Here we show that integron gene cassettes, in addition to other mobile genetic elements, appear to play a role in the mobilisation of plant symbiosis traits. This could have substantial implications for plant health and microbial community dynamics, particularly in the phyllosphere. Given the high incidence of Xanthomonadales cassettes detected among the phyllosphere, integron-mediated HGT may influence rates of *Xanthomonas*-induced diseases, such as bacterial leaf blight and other lesion-associated diseases. However, integrons may similarly be involved in spreading PGPTs that can provide protective benefits to plant leaves.

### Plant microbiomes have structurally distinct *attC* sites compared to other environments

**Figure 5:**
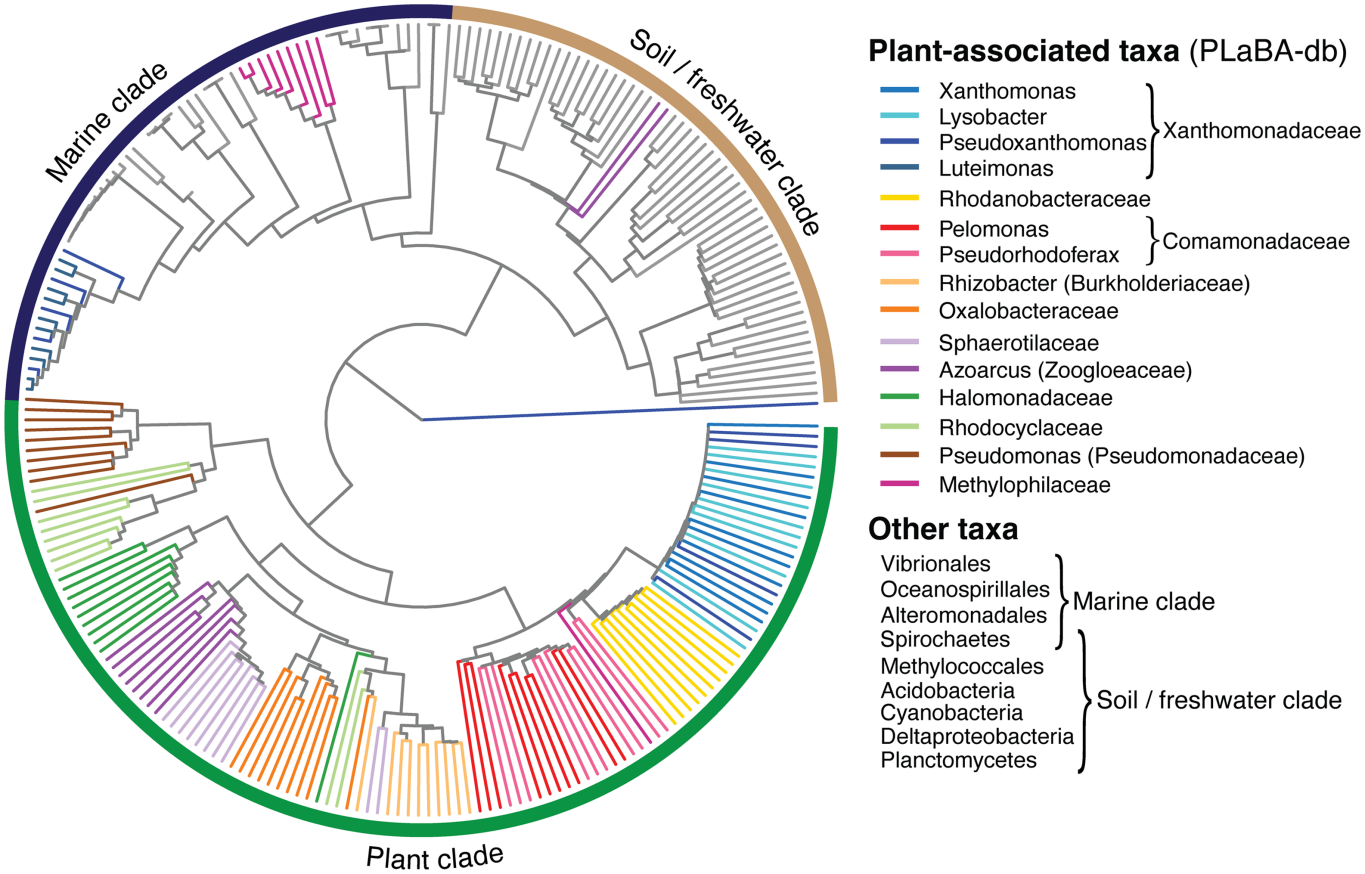
*attC* structural homology tree. Inferred relationships between representative *attC* sites obtained from plant-associated taxa (coloured branches) within PLaBA-db (Patz et al., 2021), and diverse integron-carrying bacteria (grey branches) identified in Ghaly et al. (2021b).

The single defining feature of an integron gene cassette is its recombination site (*attC*), which is essential for its insertion and excision, to and from an integron platform. The site-specificity of *attC* sites rely on their folded single-stranded structures, rather than their DNA sequence alone (Nivina et al., 2020). Therefore, to infer the relationship between *attC*s from different bacterial taxa, we clustered *attC*s based on the similarity of their secondary structures, thus generating a structure-based *attC* tree (Figure 5). Here, we show that *attC*s from almost all plant-associated taxa within PLaBA-db form their own distinct clade. This plant clade is separate from *attC*s from other bacteria, which, in turn, cluster into two sub-clades that can be loosely associated with marine and soil/freshwater taxa, respectively (Ghaly et al., 2021b). The only exceptions are the *attC*s from the PLaBA-db genomes of *Pseudoxanthomonas*, *Luteimonas*, and Methylophilaceae, which all fall within the marine *attC* sub-clade. Methylophilaceae species are more commonly found in aquatic (marine or freshwater) rather than plant-associated environments (Salcher et al., 2019), potentially explaining their *attC* placement in the marine clade. *Luteimonas* species are also more commonly associated with marine environments (Roh et al., 2008; Romanenko et al., 2013; Fan et al., 2014). It is less clear why *attC*s of *Pseudoxanthomonas*, which are associated with diverse environments (Bansal et al., 2021), fall within the marine clade. Their placement could be due to either environmental legacy, or past horizontal acquisition of an integron. Nevertheless, the vast majority of plant-associated taxa, cluster together into a single clade, indicating that plant microbiomes have structurally distinct *attC*s compared to other environments.

We also confirm that the environmental clustering of *attC* sites is not due to their host bacterial phylogeny (Supplementary Figure S2A). Comparison of the taxonomic placement between host and *attC* trees gave a maximum topological distance (nPH85) of one. Values for nPH85 can range from zero, for identical topologies, to one, for trees that share no branch bipartitions in common (Geoghegan et al., 2017). This indicates that the diversification of *attC* structures have not co-evolved along with their host bacterial genomes. Rather, this observation can be explained by (a) convergent evolution of *attC* sites independently among plant-associated bacteria, or (b) horizontal transfer of cassettes between phylogenetically distant bacteria colonising the same environment, i.e., plant surfaces. Regardless of the drivers of the observed *attC* topology, it does consequently imply that plant-associated bacteria might be more capable of exchanging gene cassettes despite their phylogenetic separation. This is because their homologous *attC* structures are more likely to be recognised by their endogenous integron integrases (IntIs), which bind to *attC*s in the folded form (Macdonald et al., 2006), thus facilitating cassette insertion. Indeed, this could explain the high frequency of cross-species HGT events observed among these isolate genomes, given that they share homologous *attC* structures.

Similarly, the inferred phylogeny of IntIs exhibits a distinct tree topology to that of their host phylogenetic tree (nPH85 = 0.83; Supplementary Figure S2B). We found that the phylogenetic placement of Xanthomondales, Burkholderiales, and Pseudomonadales IntIs (which make up the majority integron-carrying PLaBA-db isolates; Figure 1B; Supplementary Table S1) form a clade separate to all other Gammaproteobacteria (Supplementary Figure S3). Again, this might be a result of convergent evolution, or past horizontal transfer of *intI*s between these taxa. Interestingly, there is also strong incongruence between IntI and *attC* tree topologies (nPH85 = 0.94; Supplementary Figure S2C). Indeed, integron integrases and the gene cassettes that they incorporate are independent units, which have distinct evolutionary histories, between them, and their host genomes.

## Conclusion

Here, we examine the distribution, prevalence, and diversity of integrons among plant-associated bacteria. We note that there is a significant difference in the functional profiles of gene cassettes between rhizosphere- and phyllosphere-associated strains. In particular, the rhizosphere is enriched with cassettes involved in the transport and metabolism of diverse substrates, potentially assisting bacteria adapt to an environment rich in root exudates and microbial interactions.

We also show that integrons are a hotspot for cross-species gene exchange. Interestingly, the frequency of horizontal transfer of cassettes is greater in the phyllosphere, with significantly more cassette HGT events than would be expected. Many of these horizontally transmitted cassettes appear to be involved in facilitating plant-bacterial interactions. Our findings that the phyllosphere potentially exhibits elevated rates of horizontal cassette transfer may provide an ideal opportunity to promote sustainable agriculture and native ecosystem health. Specifically, by targeting the phyllosphere for bioinoculation with bacteria carrying beneficial gene cassettes relevant to leaf health, we may be able to foster their spread among leaf microbial communities. Together, these findings strongly suggest that integrons are important elements in plant microbiomes, and that they drive HGT events that facilitate plant host adaptation.

## Data availability statement

The original contributions presented in the study are included in the article/Supplementary material, further inquiries can be directed to the corresponding authors.

## Author contributions

TG: Conceptualization, Formal analysis, Writing – original draft, Writing – review & editing. MG: Conceptualization, Funding acquisition, Writing – review & editing. VR: Conceptualization, Writing – review & editing. IP: Conceptualization, Funding acquisition, Writing – review & editing. ST: Conceptualization, Funding acquisition, Writing – review & editing.
