# Supplementary material for: Horizontal gene transfer in plant microbiomes: integrons as hotspots for cross-species gene exchange
Source: Front Microbiol. 2024 Apr 29;15:1338026. doi: 10.3389/fmicb.2024.1338026 (PMC11089894; doi:10.3389/fmicb.2024.1338026)
Supplement: Supplementary file 2 [file Image_1.pdf]

Supplementary Figures

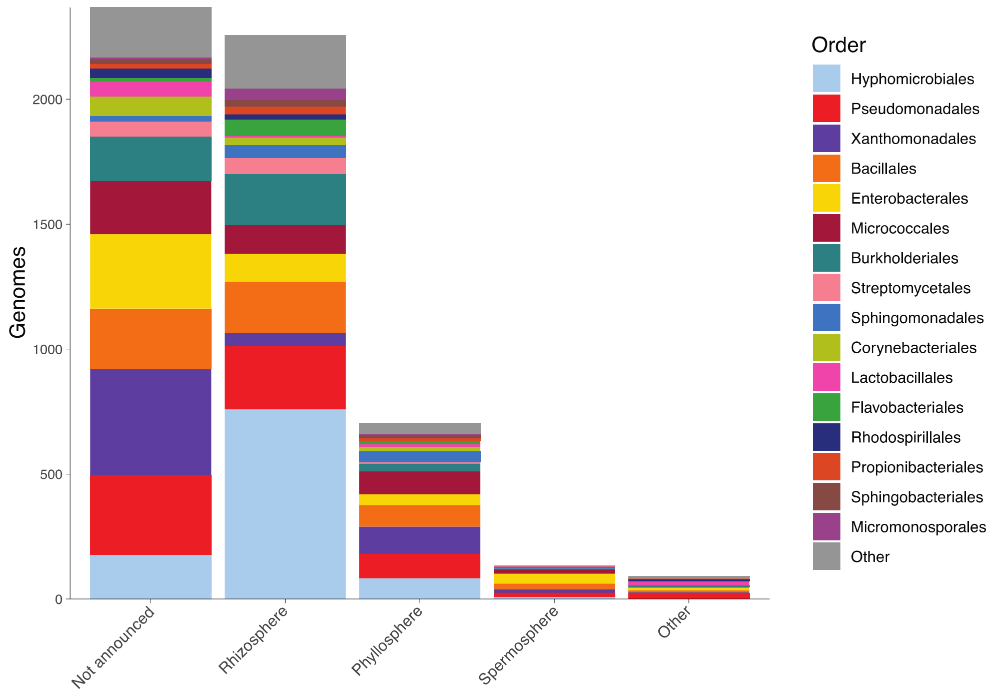

Fig. S1. Abundance and taxonomy of plant-associated bacteria by associated plant site. The stacked bar chart shows the abundance of genomes of bacteria isolated from different plant sites in the plant-associated bacterial genome database, PLaBA-db (Patz et al., 2021). Colours indicate order-level taxonomy of the isolate genomes.

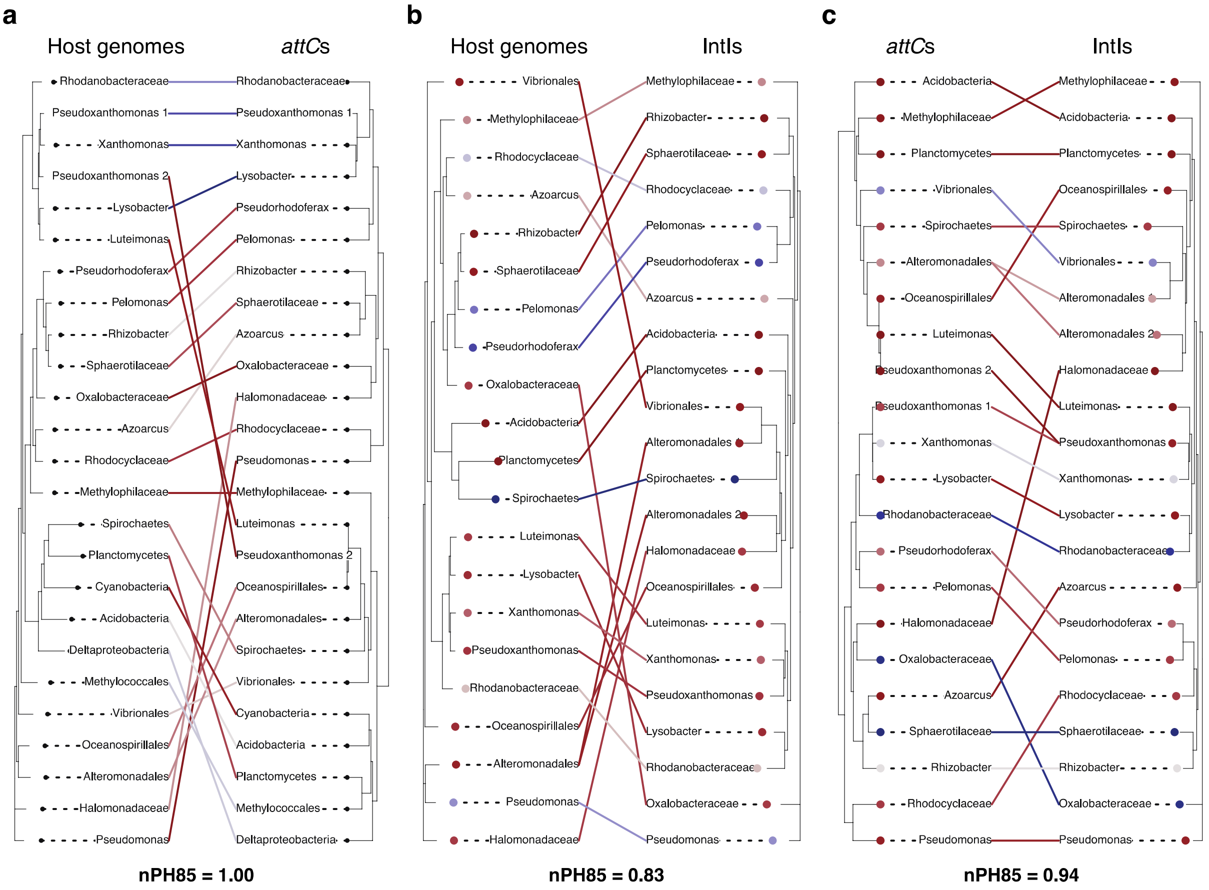

Supplementary Fig. S2. Co-phylogenetic analysis of host genome, *attC*, and IntI trees. Tanglegrams for comparing the tree topologies of **a**, host genome vs *attC* trees, **b**, host genome vs IntI trees, and **c**, *attC*s vs IntI trees. Tanglegrams were generated by rotating nodes of each tree to maximise congruence for each comparison. Matching tip labels indicate the same genome from which the *attC*, IntI or phylogenetic marker genes sequences were obtained. Lines matching the tip labels are coloured by the degree to which those tips are contributing to the topological incongruence, from highest incongruence (red) to lowest incongruence (blue). The nPH85 metric is displayed below each tanglegram, which is a quantitative measure of tree topological distance, ranging from 0.0, for identical topologies, to 1.0, for trees that share no branch bipartitions in common.

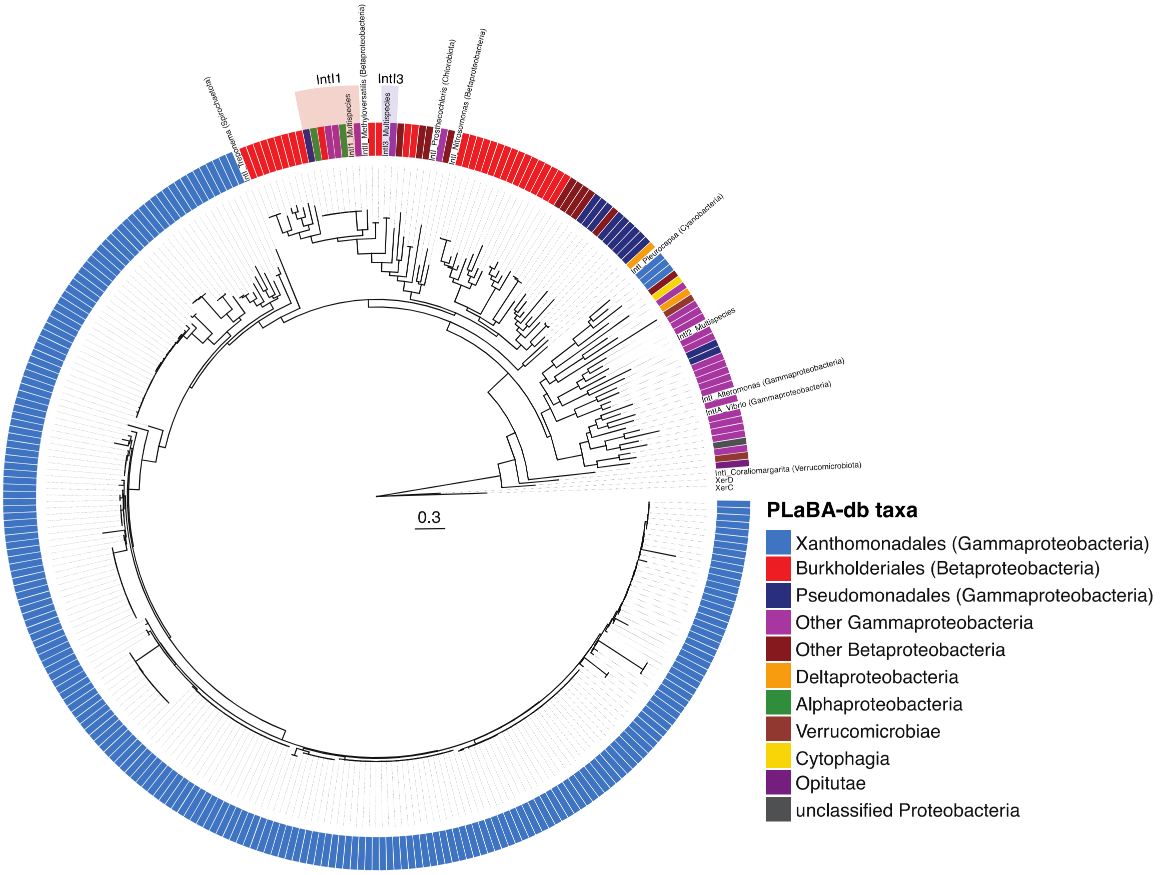

Supplementary Fig. S3. IntI phylogenetic tree. The IntI phylogeny was inferred from a maximum-likelihood approach, using Q.pfam+F+R7 as the best fit amino acid substitution model, and rooted using XerC (NP_418256.1) and XerD (NP_417370.1) as outgroup sequences. The scale bar indicates amino acid substitutions per site. The outer ring is coloured according to order-level taxonomy. Reference IntIs were included (shown as tip labels) to cover the known taxonomic diversity of integron-carrying bacteria. These encompassed the class 1, 2 and 3 IntIs, displayed as IntI1_multispecies (WP_000845048.1), IntI2_Multispecies (WP_063962748.1), and IntI3_Multispecies (AHD24676.2), respectively, as well as IntI_Treponema_Spirochaetota (WP_002679544.1), IntI_Methyloversatilis_Betaproteobacteria (WP_069040593.1), IntI_Prosthecochloris_Chlorobiota (WP_083188083.1), IntI_Nitrosomonas_Betaproteobacteria (WP_011634422.1), IntI_Pleurocapsa_Cyanobacteria (WP_015144300.1), IntI_Alteromonas_Gammaproteobacteria (WP_118490364.1), IntIA_Vibrio_Gammaproteobacteria (WP_000841999.1), and IntI_Coraliomargarita_Verrucomicrobiota (WP_280980095.1).
